# Automatic recognition of dynamic signs of Mexican sign language using deep learning

**DOI:** 10.3389/frai.2026.1794923

**Published:** 2026-04-16

**Authors:** Jesús Antonio Navarrete-López, Michelle Sainos-Vizuett, Irvin Hussein Lopez-Nava

**Affiliations:** Data Science and Machine Learning Lab, Centre for Scientific Research and Higher Education of Ensenada (CICESE), Ensenada, Baja California, Mexico

**Keywords:** deep learning, Lengua de Señas Mexicana, LSM, Mexican Sign Language, sign language recognition

## Abstract

**Introduction:**

Over four million individuals in Mexico face communication barriers due to hearing impairments. Sign language serves as an essential communication tool within the deaf community; however, automatic translation between sign and oral languages remains a significant challenge. This study proposes an approach for recognizing dynamic gestures from Mexican Sign Language (LSM) to support the development of assistive communication technologies.

**Methods:**

In collaboration with expert interpreters, an LSM corpus comprising 121 signs was developed, including a specialized lexicon focused on medical emergencies and accident scenarios. A standardized video acquisition protocol was implemented with both expert and non-expert participants. The proposed methodology consists of skeletal keypoint extraction using MediaPipe, data augmentation through frame sampling, and dataset normalization. Multiple deep learning architectures were evaluated, including ResNet, Simple RNN, LSTM, Bidirectional LSTM (BiLSTM), Gated Recurrent Units (GRU), a Transformer encoder, and a hybrid ResNet–Transformer model.

**Results:**

Among the evaluated models, the ResNet architecture achieved the best performance, obtaining an F1-score of 0.948 under subject-independent evaluation, with an average inference time of 0.468 seconds. Hyperparameter optimization analysis indicated that performance improvements were primarily driven by training dynamics and regularization strategies rather than increases in architectural depth.

**Discussion:**

The results demonstrate the effectiveness of deep learning–based approaches for dynamic LSM gesture recognition and highlight the importance of optimization strategies for robust generalization. This work contributes toward LSM-to-Spanish translation systems and provides a foundation for advancing data-driven sign language recognition technologies.

## 1 Introduction

According to the World Health Organization (WHO), 5% of the global population has hearing disabilities, a seemingly small percentage that represents 460 million individuals worldwide (Farooq et al., 2021). This number is projected to rise to 900 million by 2050. In Mexico, there are 4.2 million people with hearing disabilities; of these, 1.3 million have severe hearing loss (often considered deafness), while the remaining 2.9 million experience moderate hearing loss (Mejía-Peréz et al., 2022). Hearing loss is categorized into mild, moderate, severe, or profound, based on the degree of impairment. Individuals who are severely or profoundly deaf are often unable to hear others, leading to significant communication challenges (Martínez-Gutiérrez et al., 2019).

Sign languages serve as the primary means of communication within deaf communities worldwide. These visual-gestural languages utilize hand shapes, movements, and facial expressions, where each gesture may represent a word, or gloss, in a spoken language (Bragg et al., 2019). The official sign language in Mexico is Mexican Sign Language (LSM), which has been recognized as a national language since 2005. According to Lissi et al. (2012), the deaf population in Mexico can be categorized into four language proficiency groups:

- Monolinguals, who communicate exclusively in LSM.
- Bilinguals, comprising individuals fluent in two languages: (i) LSM as a first language, with varying proficiency in oral and/or written Spanish or another sign language; (ii) Spanish as a first language, with LSM or another sign language as a second language.
- Multilinguals, who are proficient in LSM, Spanish, and another natural language (e.g., English) or another sign language, such as American Sign Language (ASL).
- Semilinguals, who lack full competence in both LSM and Spanish, often including those without formal education who rely on home signs or family-specific gestures for communication.

This categorization is essential for understanding the communication barriers between the deaf community and the Spanish-speaking majority in Mexico. For millions, sign language is the primary medium for interacting with the world. However, most hearing individuals lack proficiency in these languages. In Mexico, an estimated 60% of the deaf population is illiterate, largely due to limited access to education. This educational gap creates profound communication obstacles, restricting educational, employment, and social opportunities for deaf individuals. Consequently, sign language recognition and translation technologies hold vast potential. Deep Neural Networks, in particular, have shown significant promise for this task. As suggested by Cui et al. (2019), their full impact on Sign Language Recognition (SLR) will continue to unfold over the coming decade.

There are over 200 distinct sign languages worldwide, each as unique as spoken languages. LSM possesses its own grammar and lexicon and is not directly related to oral Spanish (Faurot et al., 1999). It also differs considerably from Spanish Sign Language (LSE) and American Sign Language (ASL), although both LSM and LSE share historical roots in Old French Sign Language. This linguistic diversity is a critical consideration when developing sign language recognition systems, as robust datasets for many of these languages remain scarce.

**Figure 1:**
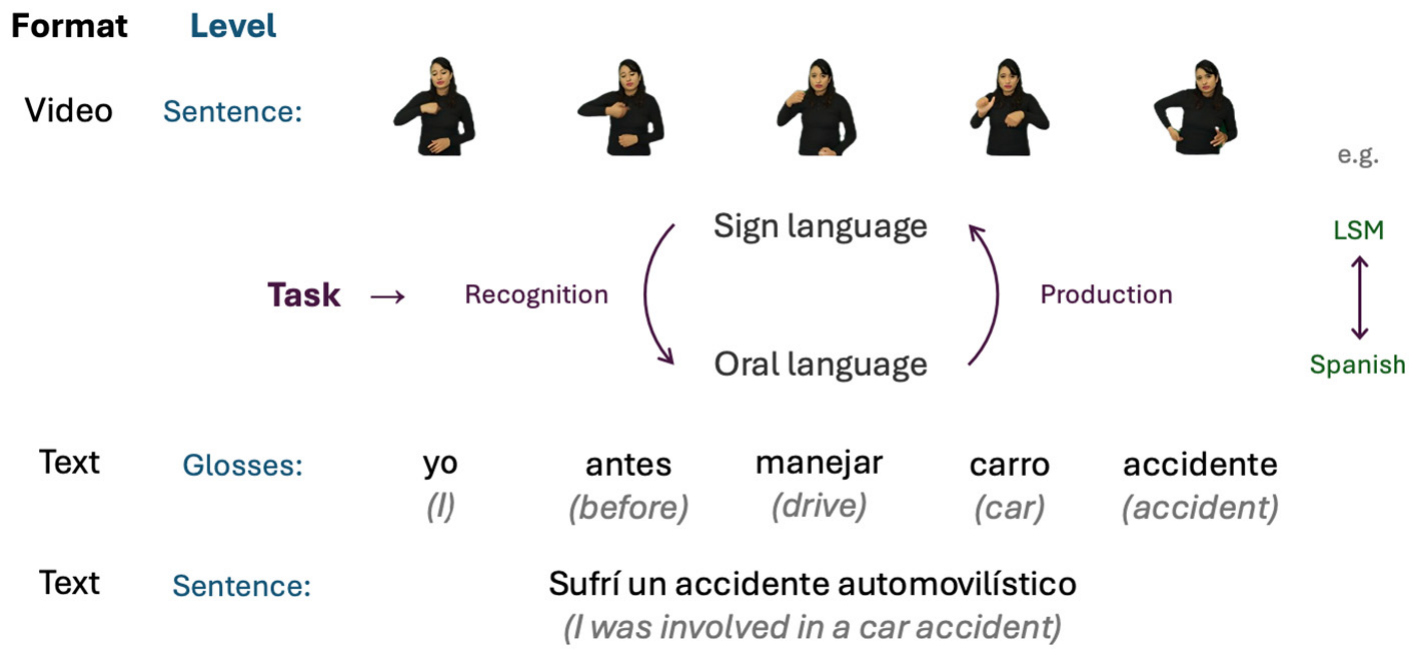
Translation between sign and oral languages.

The translation of sign languages presents a distinct challenge within natural language processing due to their visual-spatial nature, necessitating specialized methodologies. Effective translation systems must address bidirectional communication barriers between hearing and deaf individuals, requiring tailored solutions for both sign-to-text/speech and text/speech-to-sign conversion (Sainos-Vizuett, 2022); see Figure 1. Both directions are equally vital: sign-to-oral translation enables the hearing community to understand deaf individuals, while oral-to-sign translation supports deaf individuals who have limited command of the oral language.

Sign languages are agraphic, meaning they lack a standard written form. This absence complicates grammatical analysis and documentation. To address this, linguists use glosses—written transcriptions of signs—as a means to categorize and study sign language lexicon and grammar. A gloss transcribes a sign using one or more written words, providing a tangible reference for analysis.

As illustrated in Figure 1, translating between sign and oral languages requires appropriate encoding formats. Sign languages are inherently visual, so input is typically represented as images or video sequences. In contrast, output for oral languages is commonly represented as text (including glosses) or speech. Therefore, sign language translation is a multidisciplinary endeavor that integrates computer vision, human motion analysis, artificial intelligence, and natural language processing.

The rest of the paper is structured as follows. Section 2 reviews the most significant work related to the translation of Mexican Sign Language to Spanish. Section 3 describes the methodology proposed in this study. Section 4 presents the results of the sign recognition models. Finally, Section 5 discusses the conclusions and future work.

## 2 Related work

The automatic recognition of sign language, particularly the translation from Mexican Sign Language (LSM) captured in video to written Spanish, has attracted significant interest in recent years. By using cameras as the primary sensing modality, most studies approach sign recognition as a classification task, where sequences of video frames are processed to extract meaningful features. These features are subsequently used to train machine learning models that associate a sequence of frames with specific concepts or words. Various methodologies, spanning traditional machine learning and contemporary deep learning approaches, have been explored to address this challenge. This section provides a comprehensive review of relevant studies published within the last decade, emphasizing their methodological contributions and recognized limitations.

Studies in LSM recognition have employed diverse data collection strategies and input modalities. While the primary focus has been on LSM (Borges-Galindo et al., 2024; González-Rodríguez et al., 2024; Hilario-Acuapan et al., 2025; Martínez-Sánchez et al., 2023; Mejía-Peréz et al., 2022; Sosa-Jiménez et al., 2022; Martinez-Seis et al., 2019; Espejel-Cabrera et al., 2021; García-Bautista et al., 2017; Cervantes et al., 2016), some research, such as that by Miah et al. (2024), has extended evaluation to multiple sign languages, including American Sign Language (ASL), Pakistani Sign Language (PSL), and LSM. The types of signs addressed range from isolated words or glosses (dynamic or static) (Borges-Galindo et al., 2024; Hilario-Acuapan et al., 2025; Martínez-Sánchez et al., 2023; Mejía-Peréz et al., 2022; Miah et al., 2024; Sosa-Jiménez et al., 2022; Martinez-Seis et al., 2019; Espejel-Cabrera et al., 2021; García-Bautista et al., 2017; Cervantes et al., 2016) to phrases or sentences (dynamic) (González-Rodríguez et al., 2024; Hilario-Acuapan et al., 2025). Vocabulary sizes vary significantly, from as few as 10 phrases (González-Rodríguez et al., 2024) or 20 words (Borges-Galindo et al., 2024; García-Bautista et al., 2017), to extensive lexicons comprising 82 (Sosa-Jiménez et al., 2022) or 100 (Martínez-Sánchez et al., 2023). Application domains include general communication (Miah et al., 2024; Borges-Galindo et al., 2024; Mejía-Peréz et al., 2022; Martinez-Seis et al., 2019; García-Bautista et al., 2017), medical contexts (Sosa-Jiménez et al., 2022), and educational environments (González-Rodríguez et al., 2024). Data capture has predominantly been performed using RGB cameras (Borges-Galindo et al., 2024; Hilario-Acuapan et al., 2025; Martínez-Sánchez et al., 2023; Cervantes et al., 2016; Espejel-Cabrera et al., 2021), RGB-D cameras such as Microsoft Kinect v1 (Sosa-Jiménez et al., 2022; García-Bautista et al., 2017) and OAK-D (González-Rodríguez et al., 2024; Mejía-Peréz et al., 2022), as well as high-speed cameras (Hilario-Acuapan et al., 2025). Capture conditions vary from controlled laboratory settings (Sosa-Jiménez et al., 2022; Espejel-Cabrera et al., 2021; Cervantes et al., 2016) to realistic, uncontrolled environments (Martínez-Sánchez et al., 2023). Participant numbers in data collection typically range from a few individuals (Martínez-Sánchez et al., 2023) to 35 deaf signers (García-Bautista et al., 2017), often including deaf and hearing individuals, as well as expert and non-expert signers, ensuring diverse representation (Sosa-Jiménez et al., 2022; García-Bautista et al., 2017; Cervantes et al., 2016; Hilario-Acuapan et al., 2025).

Preprocessing often involves extracting skeletal keypoints using tools such as MediaPipe Holistic (Borges-Galindo et al., 2024; Mejía-Peréz et al., 2022), OpenPose (Miah et al., 2024), or YOLOv8-pose (Hilario-Acuapan et al., 2025), capturing body, hand, and facial postures. Some approaches directly process raw video data (Martínez-Sánchez et al., 2023), while others utilize segmented hand images (Sosa-Jiménez et al., 2022) or geometric/color features (Espejel-Cabrera et al., 2021; Cervantes et al., 2016). Common preprocessing techniques include spatial normalization of keypoint coordinates (Miah et al., 2024; Mejía-Peréz et al., 2022; Sosa-Jiménez et al., 2022; Cervantes et al., 2016), trajectory smoothing using LOESS regression (Sosa-Jiménez et al., 2022), and filtering inconsistent data (Miah et al., 2024). Segmentation methods range from color-based neural networks for skin detection (Espejel-Cabrera et al., 2021) to K-means clustering for image segmentation (Martinez-Seis et al., 2019). Feature extraction frequently involves selecting specific subsets of keypoints (González-Rodríguez et al., 2024; Mejía-Peréz et al., 2022), or focusing on specific attributes such as arm movements (Hilario-Acuapan et al., 2025), invariant vectors derived from hand trajectories (García-Bautista et al., 2017), or geometric and color descriptors (Cervantes et al., 2016). Dimensionality reduction has also been applied, notably using genetic algorithms for feature selection (Cervantes et al., 2016).

The models employed for LSM recognition encompass various architectures. Convolutional Neural Networks (CNNs), such as MobileNet (Martinez-Seis et al., 2019) and YOLOv8-based classifiers (Hilario-Acuapan et al., 2025), are frequently used. Recurrent Neural Networks (RNNs), including long short-term memory (LSTM), Bidirectional LSTM (BiLSTM), and Gated Recurrent Units (GRU), are widely adopted due to their sequential data handling capabilities (Borges-Galindo et al., 2024; González-Rodríguez et al., 2024; Martínez-Sánchez et al., 2023; Mejía-Peréz et al., 2022). Advanced approaches incorporate Graph Convolutional Networks (GCNs) to model spatial relationships between keypoints (Miah et al., 2024). Hybrid models and ensembles are common, exemplified by the two-stream Graph Convolutional Attention Residual Network (GCAR) (Miah et al., 2024), and models combining pre-trained CNNs with LSTMs (Martínez-Sánchez et al., 2023). Hidden Markov Models (HMMs) have been utilized in bidirectional recognition and synthesis systems (Sosa-Jiménez et al., 2022). Traditional machine learning methods, including Support Vector Machines (SVM) (Cervantes et al., 2016; Espejel-Cabrera et al., 2021) and Dynamic Time Warping (DTW) (García-Bautista et al., 2017), have demonstrated robust performance. Common optimization strategies include the Adam optimizer (Miah et al., 2024), regularization methods such as Dropout (Miah et al., 2024), residual connections (Miah et al., 2024), attention mechanisms (Miah et al., 2024; González-Rodríguez et al., 2024), and early stopping (Miah et al., 2024). The Baum-Welch algorithm has been specifically applied for HMM training (Sosa-Jiménez et al., 2022). Performance evaluations typically include metrics such as Top-K accuracy (Miah et al., 2024; Hilario-Acuapan et al., 2025), overall accuracy, sensitivity, specificity, precision, and F1-score (Sosa-Jiménez et al., 2022). Several studies report high accuracy exceeding 99% on their datasets (Martínez-Sánchez et al., 2023; García-Bautista et al., 2017), while others achieve commendable results ranging from 0.81 to 0.93 (Borges-Galindo et al., 2024). Common errors in classification are frequently due to spatial similarities between signs (Miah et al., 2024), reduced hand visibility, or rapid movements (Sosa-Jiménez et al., 2022; Martinez-Seis et al., 2019; Hilario-Acuapan et al., 2025).

Despite these notable advancements, several challenges and limitations persist in the existing literature, which our proposed study aims to address. A common limitation is the difficulty in effectively handling a very large number of classes or extensive vocabularies, where model accuracy tends to decrease (Miah et al., 2024). This indicates a need for more scalable architectures or hierarchical classification strategies. The generalization capability of models remains a significant concern, as many studies rely on datasets with a limited number of participants (Martínez-Sánchez et al., 2023; Mejía-Peréz et al., 2022; Sosa-Jiménez et al., 2022; García-Bautista et al., 2017) or controlled capture conditions (Sosa-Jiménez et al., 2022; Espejel-Cabrera et al., 2021; Cervantes et al., 2016), which can hinder robust performance in uncontrolled environments or with unseen signers (Cervantes et al., 2016). Regional variations in LSM and differences in non-expert facial expressions also pose challenges to generalization (Martínez-Sánchez et al., 2023). Furthermore, inherent sensor limitations, including noise (Miah et al., 2024; Mejía-Peréz et al., 2022), vulnerability to lighting changes (Sosa-Jiménez et al., 2022), and hardware issues like overheating (Sosa-Jiménez et al., 2022), continue to affect system reliability. The complexity of signs, particularly those involving high-speed movements, occlusions (Sosa-Jiménez et al., 2022; Martinez-Seis et al., 2019; Hilario-Acuapan et al., 2025), or subtle spatial differences (Miah et al., 2024), often leads to misclassifications. Additionally, some systems may focus exclusively on specific features (e.g., arm movement) or exclude complex non-manual signs (González-Rodríguez et al., 2024; Hilario-Acuapan et al., 2025), limiting a comprehensive understanding of LSM. Lastly, data scarcity is a pervasive issue, highlighting the continuous need for larger, more diverse, and representative datasets that capture real-world conditions (Hilario-Acuapan et al., 2025; Martínez-Sánchez et al., 2023). Many existing works primarily focus on isolated words, presenting scalability challenges for the recognition of continuous sign language and complex grammatical structures (Borges-Galindo et al., 2024; García-Bautista et al., 2017).

To address these limitations, the present work proposes a specialized corpus that encompasses frequently used signs alongside a dedicated lexicon for medical emergencies and accident scenarios, developed and validated in collaboration with a regional association of deaf individuals. This corpus emphasizes collaboration with expert and non-expert signers to enhance data representativeness and involves evaluating deep learning models to increase robustness against the intrinsic variability of LSM contexts.

## 3 Methods

**Figure 2:**
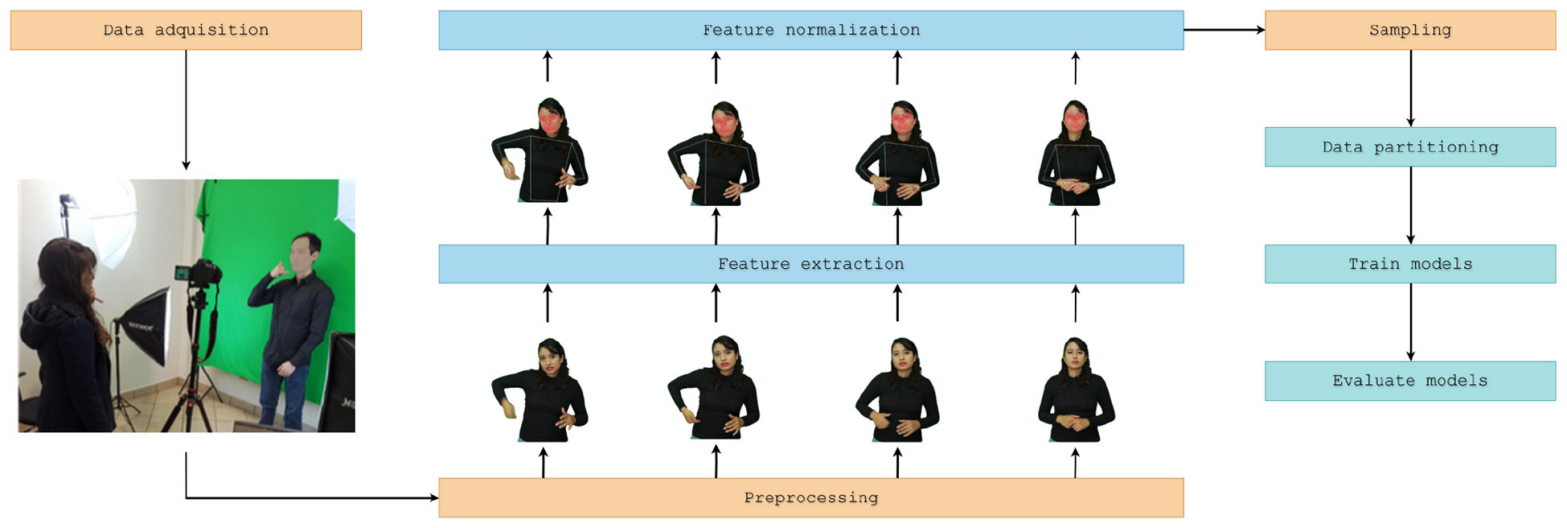
Proposed methodology.

To develop an automatic recognition system for Mexican Sign Language (LSM), a structured supervised learning methodology was followed, as summarized in Figure 2. In collaboration with LSM experts, 121 relevant glosses were identified within the context of medical emergencies and accident scenarios. Subsequently, 2D video sequences corresponding to each identified gloss were recorded.

Once the sequences were collected, Google's MediaPipe Holistic was employed to extract skeletal keypoints representing the body, hands, and face from each video frame. The extracted data underwent a preprocessing stage involving feature scaling for normalization and a strategy for handling missing data, ensuring dataset quality and consistency.

After preprocessing, the resulting dataset was used to train multiple deep learning (DL) models with different architectures, aiming to accurately classify each sign based on extracted patterns. Finally, the trained models were evaluated using metrics assessing both classification accuracy and computational efficiency.

### 3.1 Data capturing

The data collection process was meticulously designed under the supervision of expert LSM interpreters. This collaboration enabled the construction of a well-defined and contextually relevant corpus focused on emergency medical and accident scenarios involving deaf individuals. To ensure consistency and reliability during data acquisition, a structured recording protocol was implemented. The sessions were conducted in a controlled environment, and the participant pool included 10 non-expert individuals and two expert LSM signers, each instructed to perform the designated signs according to the defined protocol. Given that the recordings included participants' facial features as part of the visual modality, the study was reviewed and approved by an ethics committee (approval ID: CICESE_HUM_2021_07).

#### 3.1.1 Sign vocabulary design

Through expert collaboration, a total of 110 Spanish phrases were initially proposed, covering medical emergencies and accident, common symptoms, and polite expressions relevant to healthcare interactions. Expert supervision was essential for refining the dataset by eliminating phrases that were either contextually inappropriate for deaf individuals or semantically redundant. During this refinement process, 10 phrases were excluded. For example:


“Tengo escurrimiento nasal” (*I have nasal discharge*)
“Tengo síntomas de gripe” (*I have flu symptoms*)


Although these phrases carry distinct meanings in spoken Spanish, they share an identical interpretation in LSM. Additionally, phrases subject to regional variation—where expert signers offered inconsistent interpretations—were also removed to preserve consistency and reduce ambiguity within the dataset.

**Figure 3:**
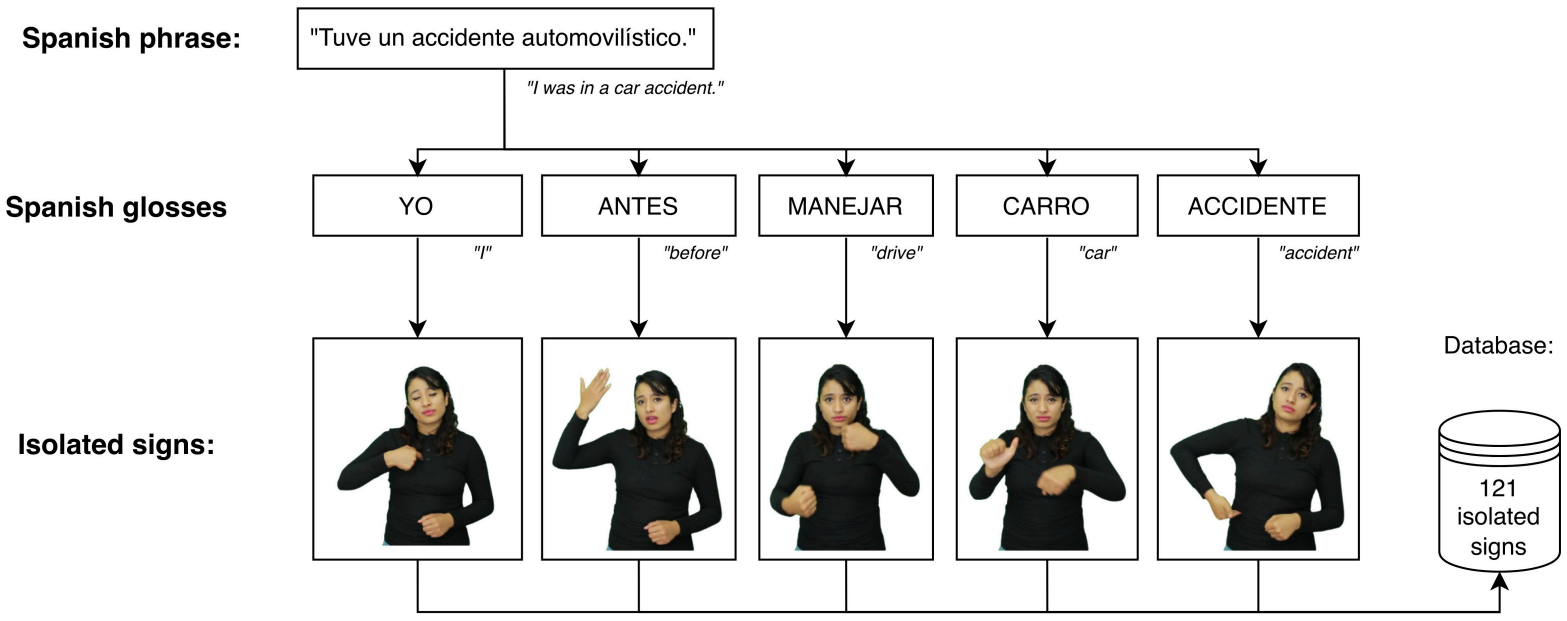
Process of decomposing phrases into glosses.

Subsequently, the sentences were decomposed into glosses in order to isolate individual signs and construct a dataset of isolated signs for the automatic recognition task. An example of this process is illustrated in Figure 3, where the Spanish sentence “Tuve un accidente automovilístico” (“I had a car accident”) is decomposed into its corresponding Spanish gloss sequence: YO, ANTES, MANEJAR, CARRO, ACCIDENTE (I, before, drive, car, accident). This gloss-based decomposition facilitated the development of a linguistic model that guided the data acquisition process, ensuring that each lexical unit was captured in a controlled and consistent manner.

#### 3.1.2 Capturing process

Given the challenges involved in recruiting expert deaf LSM signers, an alternative methodology was implemented. Instructional videos featuring (linguistic model) LSM experts performing each sign were recorded in advance, allowing non-expert participants to replicate the gestures with guidance. To enhance the precision of the feature-extraction system and minimize false positives, all recordings were carried out in a controlled environment. The capture setup included:

- A green screen (chroma key) background to facilitate keypoint extraction.
- A Nikon D5500 (Nikon Corporation, Tokyo, Japan) camera recording at 1,920 × 1,080p resolution and 60 FPS.
- A multi-point lighting system to eliminate shadows and improve sign visibility.

To ensure the reliability of the data collection process, a pilot study was conducted prior to full-scale recording. In this preliminary phase, participants were shown the instructional videos and asked to replicate each sign slowly to standardize execution. The final data collection followed a structured protocol:

1. Participants received a briefing on the correct articulation of each sign, including the roles of the dominant and supporting hand.
2. Each participant watched an instructional video of the target sign.
3. Participants practiced the sign twice before the recording.
4. The sign was recorded at 60 frames per second (fps), with each video lasting between 2 and 4 s.
5. Steps 2–4 were repeated for each sign, with a 5-min rest period after every 50 signs to reduce fatigue.

In addition, participants were instructed to wear dark, long-sleeved clothing to maximize contrast against the green screen. Each session lasted approximately 2 h, ensuring the complete acquisition of the predefined sign vocabulary.

### 3.2 Preprocessing

Once the data collection phase was finalized, the preprocessing workflow was initiated. This included keypoint extraction from the videos, followed by data augmentation, normalization, and the handling of missing values.

#### 3.2.1 MediaPipe holistic

A framework developed by Google enables real-time detection and tracking of multiple regions of the human body, including the face, hands, and upper body posture. It integrates several inference models into a unified pipeline, ensuring efficient and coherent capture of anatomical keypoints. The model produces a total of 543 keypoints distributed across three primary regions:

- Pose estimation: 33 keypoints optimized for skeletal detection.
- Hand tracking: 21 keypoints per hand, providing detailed information on finger structure and orientation.
- Facial landmarks: 468 keypoints enabling precise characterization of facial expressions and motion.

Given the nature of the recorded videos, facial landmarks were excluded from the dataset, as the non-expert participants did not consistently reproduce meaningful or semantically accurate facial expressions. Furthermore, from the 33 pose estimation keypoints, only those corresponding to the upper body (keypoints 0 to 14) were retained for analysis.

#### 3.2.2 Data sampling

To address the limited availability of training samples—since each sign has only one video per subject—the Frame Skip Sampling technique proposed by Ko et al. (2019) was employed. This method systematically and randomly selects subsets of frames from the original video sequences, allowing the generation of multiple augmented sequences. Given a video sequence shown in Equation 1.


$$\begin{array}{l} S=\left(f_{1},f_{2},...,f_{l}\right) \end{array}$$ (1)

with *l* frames, a fixed number of frames is randomly selected, in this case, *n* = 15. The average spacing between frames is computed as shown in Equation 2:


$$\begin{array}{l} z=\left\lfloor \frac{l}{n-1}\right\rfloor \end{array}$$ (2)

Next, a set of frames is extracted using the indices defined in Equation 3:


$$\begin{array}{l} Y=\left(y,y+z,y+2z,...,y+\left(n-1\right)z\right) \end{array}$$ (3)

where *y*, defined in Equation 3, is computed as $y=\left\lfloor \frac{l-z\left(n-1\right)}{8}\right\rfloor$ is referred to as the base sequence. Subsequently, a sequence of random numbers is generated as shown in Equation 4:


$$\begin{array}{l} R=\left(r_{1},r_{2},...,r_{n}\right) \end{array}$$ (4)

with values ranging from [1, *z*]. A new sequence *Y*_*new*_ is then obtained by summing the base sequence with the random sequence as defined in Equation 5:


$$\begin{array}{l} Y_{new}=\left(Y_{1}+R_{1},Y_{2}+R_{2},...,Y_{n}+R_{n}\right) \end{array}$$ (5)

In this study, 50 new sequences of *n* = 15 frames were generated from each original video. As a result, the final dataset comprises 500 samples per sign class in the proposed vocabulary.

#### 3.2.3 Data normalization

To mitigate inconsistencies in hand positioning across different video sequences, it is crucial to standardize the spatial distribution of keypoints. For this purpose, a two-dimensional feature vector is constructed as defined in Equation 6:


$$\begin{array}{l} V=\left(v_{1},v_{2},\ldots ,v_{n}\right)\in {\mathbb{R}}^{n\times 2} \end{array}$$ (6)

where each element $v_{i}=\left(v_{i}^{x},v_{i}^{y}\right)$ corresponds to a specific hand keypoint.

To facilitate normalization, two separate vectors are derived as shown in Equation 7:


$$\begin{array}{l} V_{x}=\left(v_{1}^{x},v_{2}^{x},\ldots ,v_{n}^{x}\right),\;V_{y}=\left(v_{1}^{y},v_{2}^{y},\ldots ,v_{n}^{y}\right) \end{array}$$ (7)

representing the keypoints' coordinates along the *x* and *y* axes, respectively.

Normalization is performed using *z*-score standardization, ensuring that the transformed coordinates have zero mean and unit variance. The transformation for the *x*-coordinate vector is defined in Equation 8:


$$\begin{array}{l} V_{x}^{*}=\frac{V_{x}-\mu \left(V_{x}\right)}{\sigma \left(V_{x}\right)} \end{array}$$ (8)

where μ(*V*_*x*_) denotes the mean and σ(*V*_*x*_) represents the standard deviation of *V*_*x*_. A similar procedure is applied to the *y*-coordinate vector, yielding Equation 9:


$$\begin{array}{l} V_{y}^{*}=\frac{V_{y}-\mu \left(V_{y}\right)}{\sigma \left(V_{y}\right)} \end{array}$$ (9)

Thus, the final standardized feature representation is given in Equation 10:


$$\begin{array}{l} V^{*}=\left[V_{x}^{*},V_{y}^{*}\right]\in {\mathbb{N}}^{2n} \end{array}$$ (10)

This normalization process ensures that keypoints remain invariant to scale and positioning differences, enhancing the robustness of the feature representation across varying conditions.

#### 3.2.4 Handling missing data

To address missing values within the dataset, a forward-fill imputation strategy was implemented across the temporal dimension of each video sequence. Given that each frame contains a set of keypoints associated with different body parts (e.g., pose, right hand, left hand), it is crucial to ensure continuity in keypoint trajectories to prevent disruptions in the model's learning process.

The implemented approach performs a frame-wise forward fill by propagating the last observed value for each keypoint-axis combination across consecutive frames. Specifically, for each body part *b* and its corresponding set of keypoints *K*_*b*_, the missing values are filled using the following procedure:

1. For each keypoint, the coordinates in the *X* and *Y* axes were identified across *n* = 15 consecutive frames.
2. If a missing value was found in a frame, the most recent available value from previous frames was used to fill the gap.
3. The same process was applied independently to both the *X* and *Y* coordinates of each keypoint.
4. Once all missing values were filled, the updated information was reintegrated into the dataset.

This ensures that missing keypoints are smoothly propagated over time, reducing the impact of tracking failures during video processing.

#### 3.2.5 Data partitioning

To evaluate model performance, a Leave-One-Out Cross-Validation (LOOCV) strategy was implemented. This procedure involved a total of 10 iterations, where in each iteration, data from one non-expert subject were excluded from training and used exclusively as the test set. The remaining data were randomly partitioned into 90% for training and 10% for validation. This subject-independent evaluation protocol ensures that each model is tested on data from an unseen participant, thereby providing a reliable assessment of generalization ability and robustness across subjects.

### 3.3 Classification models

Several deep learning architectures were implemented to classify LSM sign sequences. These models were designed to process keypoint-based representations and leverage the temporal structure inherent to sign gestures. The evaluated architectures include recurrent models such as Simple RNN, LSTM, BiLSTM, and GRU to capture temporal dependencies, as well as convolutional and attention-based approaches, including a 1D ResNet architecture for temporal feature extraction, a Transformer encoder model for global sequence modeling, and a hybrid CNN–Transformer architecture (ResNet–Transformer) combining convolutional feature learning with self-attention mechanisms. Each model was adapted to maximize classification performance while maintaining computational efficiency. The input was reshaped to (15, 114), where 15 denotes the sequence length (frames) and 114 corresponds to the spatial coordinates (*x* and *y*) of 57 keypoints. To ensure a fair comparison across architectures, all models were trained for a fixed number of 25 epochs during the evaluation stage.

#### 3.3.1 One-dimensional residual network (1D ResNet)

The model consists of a series of residual blocks, which allow information to propagate through deep layers using skip connections. This strategy is able to mitigate the evanescent gradient problem in deep networks, improving the learning capability of the model. Each residual block is structured as follows:

1. First convolution: a Conv1D layer with *k* = 128 filters and a kernel of *s* = 2, followed by Batch Normalization and a ReLU activation function.
2. Second convolution: the same convolutional operation is repeated, and the result is summed with the block input via a skip connection.
3. Normalization and activation: another batch normalization is applied, followed by a ReLU activation function.
4. Dimensionality reduction: a MaxPooling1D operation with a window size of 2 and a stride of 2 is added to reduce the data passed to the next layer.

The full network comprises three residual blocks, preceded by an initial Conv1D layer. The final features are flattened into a one-dimensional vector using a Flatten layer. This representation is then processed by two dense layers with 128 units and ReLU activation, each followed by an 80% dropout layer to prevent overfitting. Classification is performed using a softmax output layer with as many neurons as the number of sign classes.

#### 3.3.2 Recurrent Neural Networks (RNN)

This model employs a Simple RNN layer with 128 units, processing input sequences of shape (*n, d*), where *n* = 15 represents the number of frames per sequence and *d* corresponds to the dimensionality of the extracted keypoint features. The classification module consists of two fully connected layers with 128 and 32 units, along with a 30% dropout layer. The final classification is performed via a softmax output layer.

#### 3.3.3 Long short-term memory (LSTM)

Two LSTM-based architectures were implemented to capture temporal dependencies within sign sequences, improving classification accuracy. The first model comprises a single LSTM layer with 111 units and ReLU activation, followed by an 80% dropout layer. The extracted features are processed through a fully connected layer of 128 units with an additional 70% dropout layer, concluding with a softmax output layer.

The second model, adapted from Samaan et al. (2022), employs a deeper hierarchical structure with three LSTM layers (64, 128, and 64 units, respectively) configured to retain sequential dependencies via return sequences. The classification module consists of two dense layers (64 and 32 units) followed by a softmax output layer.

#### 3.3.4 Bidirectional long short-term memory (BiLSTM)

A BiLSTM model, inspired by Samaan et al. (2022), was implemented to enhance temporal feature extraction by processing sequences bidirectionally. The architecture consists of three stacked BiLSTM layers: the first with 128 units, the second with 256, and the third with 128 units, ensuring robust sequential feature learning. The classification module contains two dense layers (64 and 32 units) followed by a softmax output layer.

#### 3.3.5 Gated recurrent units (GRU)

Following the approach of Samaan et al. (2022), a GRU model was implemented as a computationally efficient alternative to LSTMs. The model consists of three stacked GRU layers: 64 units in the first layer, 128 in the second, and 64 in the third. The classification module comprises two fully connected layers (64 and 32 units) followed by a softmax output layer.

#### 3.3.6 Transformer

A Transformer encoder architecture was employed to model temporal dependencies within sign language sequences. The model design was inspired by the encoder component proposed in Attention Is All You Need (Vaswani et al., 2017), but adapted to an encoder-only configuration focused on sequence classification rather than sequence-to-sequence generation. This design enables the extraction of contextual temporal representations suitable for sign recognition tasks. The model receives sequences of feature vectors, which are first projected into a latent embedding space of dimension *d*_model_ = 128 through a fully connected linear layer and subsequently scaled by $\sqrt{d_{\text{model}}}$ to stabilize training. A learnable classification token (CLS token) is prepended to the sequence to aggregate global contextual information via self-attention, serving as the final sequence representation for classification. To encode temporal order, sinusoidal positional encodings are added to the embeddings, supporting sequences of up to 15 frames. The representations are then processed by a stack of four Transformer encoder layers, each composed of multi-head self-attention with eight attention heads, a position-wise feed-forward network with dimensionality 256 and ReLU activation, residual connections followed by layer normalization, and dropout regularization with a rate of 0.1 applied after both attention and feed-forward operations. Self-attention is implemented using scaled dot-product attention, enabling the modeling of long-range temporal relationships between frames. After the encoder stack, only the CLS token representation is retained and passed to a classification head consisting of a fully connected layer with ReLU activation, followed by dropout and a final softmax layer that outputs class probabilities.

#### 3.3.7 ResNet-transformer

To enhance temporal feature representation, a hybrid ResNet–Transformer architecture was implemented by combining the previously described 1D ResNet backbone with the Transformer encoder introduced before. The ResNet1D architecture remains identical to the configuration described earlier; however, instead of flattening the extracted feature maps for direct classification, the convolutional features are projected through a fully connected layer with 128 units. This projection preserves the temporal structure of the sequence while adapting the feature dimensionality to match the Transformer embedding space. The resulting sequence of embeddings is then provided as input to the same Transformer encoder architecture previously described, without architectural modifications. Consequently, the encoder maintains four layers, an embedding dimension of *d*_model_ = 128, eight attention heads, a feed-forward dimension of 256, sinusoidal positional encoding for sequences of up to 15 frames, and a dropout rate of 0.1. By replacing the flattening operation with a learned dense projection, the model enables the Transformer to operate over temporally structured convolutional representations rather than globally aggregated features. This hybrid design allows local temporal patterns extracted by the convolutional backbone to be further modeled through global self-attention mechanisms. The final classification stage remains unchanged, using the CLS token representation followed by the same classification head defined in the previous model.

### 3.4 Hyperparameter optimization

After the comparative evaluation of the proposed architectures, the best-performing model was selected for futher refinement through hyperparameter optimization. This stage aimed to identify an optimal configuration while preserving generalization across subjects and avoiding manual parameter tuning.

Hyperparameter optimization was conducted using the Optuna framework (Akiba et al., 2019), which performs efficient sampling of the search space through sequential model-based optimization. The optimization process explored both architectural and training-related parameters of the 1D ResNet model. The evaluated hyperparameters included the number of the convolutional filters, kernel sizes, number of residual blocks, depth of dense layers, activations functions, dropout rates, learning rate, batch size, and number of training epochs. The complete search space is summarized in Table 1.

**Table 1:** Search space configuration for hyperparameter optimization.

Hyperparameter	Search space
Activation_i	Categorical {relu, leaky_relu, elu, gelu, silu}
Batch_size	Categorical {32, 64}
Dense_units_i	Categorical {64, 128, 256}
Dropout_i	Float [0.0, 0.4]
Epochs	Categorical {25, 50, 75}
Filters	Categorical {32, 64, 128}
Filters_1	Categorical {32, 64, 128}
Kernel_size_1	Integer [2, 5]
Kernel_size_resblocks	Integer [2, 5]
lr	Log-uniform [1e-5, 1e-2]
n_dense_layers	Integer [1, 4]
n_resblocks	Integer [1, 3]
Use_pool_i	Categorical {true, false}

The hyperparameter search employed a conditional search space where dense-layer parameters and pooling operations were dynamically defined according to the sampled architecture depth. In particular, pooling operations within residual blocks and the configurations of fully connected layers were activated only when the corresponding architectural components were selected during each trial, allowing flexible exploration of model complexity.

To ensure subject-independent evaluation, the optimization objective was defined as the average macro F1-score computed under a leave-one-subject-out evaluation protocol. For each trial, one subject was used for testing, a fixed subject was used for validation, and the remaining subjects were used for training. Performance scores obtained across all subject splits were averaged to produce the final objective value, as defined in Equation 11:


$$\begin{array}{l} \text{Score}=\frac{1}{S}\overset{S}{\underset{i=1}{\sum }}F1_{\text{macro}}\left(i\right), \end{array}$$ (11)

where *S* denotes the total number of evaluated subjects.

The optimization process consisted of 100 trials, and the configuration that obtained the highest target score was selected as the optimized model configuration for further analysis. All the experiments were implemented using the Tensorflow library developed by Google, Optuna framework for the Hyperparameter Optimization and were conducted on a workstation equipped with an NVIDIA GeForce RTX 3090 GPU (NVIDIA Corporation, Santa Clara, CA, USA) with 24 GB of VRAM, 64 GB of system RAM, and an AMD Ryzen 9 5900X (Advanced Micro Devices, Inc., Santa Clara, CA, USA) processor.

## 4 Results

### 4.1 Architecture performance comparison

**Figure 4:**
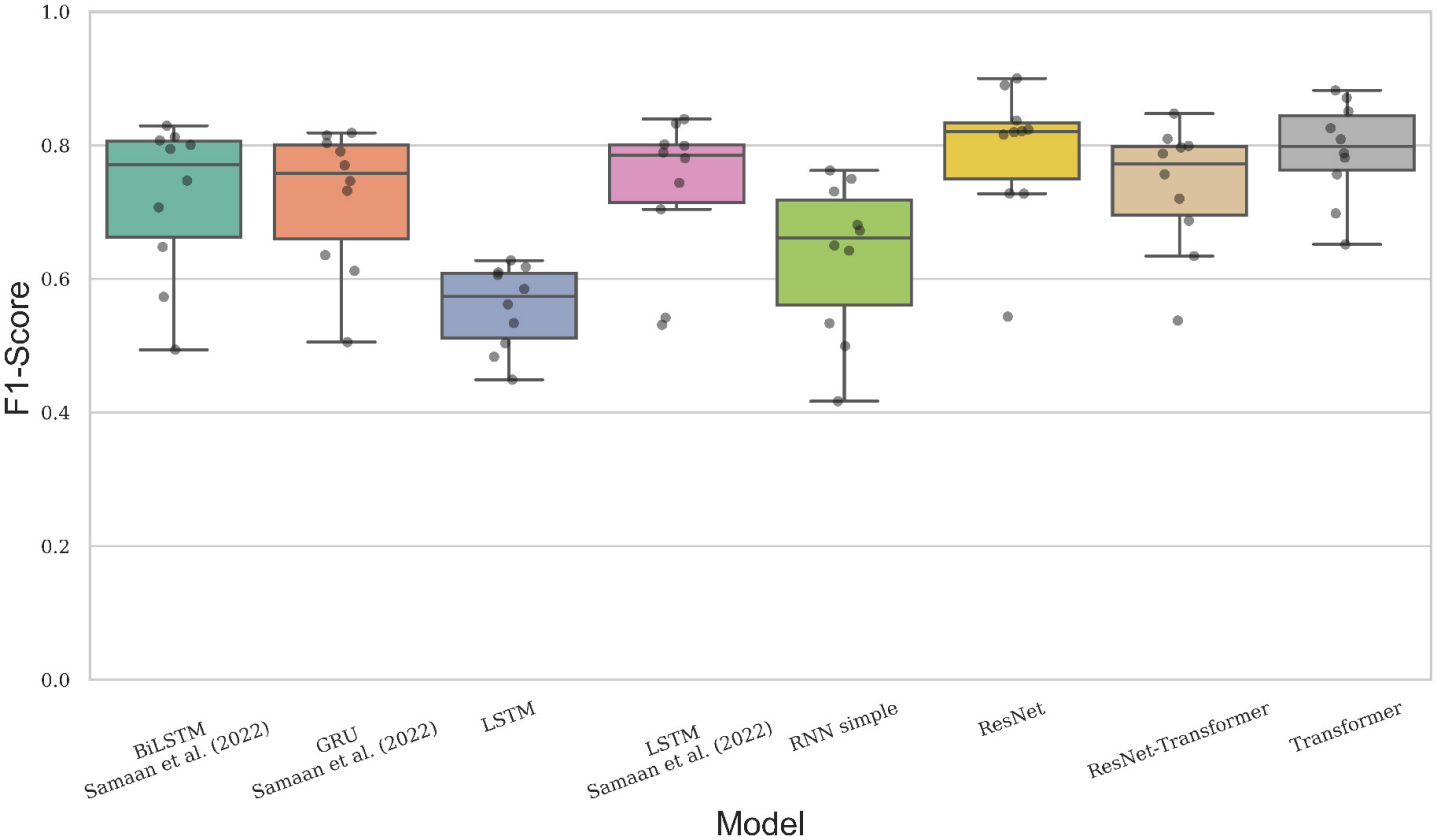
Summary results of efficiency evalutation.

The models were trained on a total of 66,570 instances, comprising 121 classes. Figure 4 presents a boxplot summarizing the results under the F1-score metric for the evaluated deep learning architectures described in Section 3.3. The ResNet-based model achieved the highest median performance, above 0.8, with low dispersion and several iterations reaching values close to 0.9, except for a single run below 0.6. The Transformer model obtained a median F1-score of approximately 0.8, demonstrating competitive performance with relatively stable behavior across runs. In contrast, the hybrid ResNet–Transformer architecture achieved a median slightly below 0.8 and, similarly to the ResNet model, presented an outlier case in which performance dropped below 0.6. The Simple RNN obtained a median close to 0.7 but exhibited higher variability, including multiple runs yielding values near or below 0.5. The single-layer LSTM recorded the lowest median performance, below 0.6, with most results concentrated between 0.5 and 0.6.

The architectures adapted from Samaan et al. (2022) displayed comparable median values above 0.7. The multi-layer LSTM showed a distribution concentrated near 0.8, similar to the BiLSTM, although the latter presented outliers below 0.6. The GRU-based model achieved some results above 0.8, but its remaining values were more widely dispersed.

Overall, these results suggest a certain degree of overfitting for specific data subsets, particularly for subjects 2 and 8, which exhibited lower recognition performance across all models. Given the large number of classes, we assessed model performance using both strict and flexible evaluation criteria, employing the Top-k accuracy metrics with Top-k = 3 and Top-k = 10. Table 2 presents these results for all evaluated models.

**Table 2:** Results of the performance evaluation (mean ± std).

Model	Accuracy	Top-3 Acc.	Top-10 Acc.	Loss	Sensitivity	Specificity	F1-score
ResNet	0.823 ± 0.093	0.916 ± 0.083	0.965 ± 0.058	1.195 ± 1.020	0.823 ± 0.093	0.998 ± 0.001	0.791 ± 0.104
Transformer	0.828 ± 0.061	0.942 ± 0.05	0.986 ± 0.017	1.197 ± 0.732	0.826 ± 0.064	0.999 ± 0.001	0.792 ± 0.074
ResNet-transformer	0.782 ± 0.08	0.923 ± 0.06	0.977 ± 0.025	1.442 ± 0.855	0.779 ± 0.088	0.998 ± 0.001	0.738 ± 0.095
RNN simple	0.677 ± 0.106	0.848 ± 0.096	0.931 ± 0.086	3.307 ± 3.09	0.677 ± 0.106	0.997 ± 0.001	0.634 ± 0.115
LSTM	0.612 ± 0.052	0.788 ± 0.067	0.928 ± 0.07	2.266 ± 2.426	0.612 ± 0.052	0.997 ± 0.0	0.558 ± 0.062
LSTM (Samaan et al., 2022)	0.774 ± 0.097	0.879 ± 0.084	0.945 ± 0.065	2.916 ± 3.073	0.774 ± 0.097	0.998 ± 0.001	0.737 ± 0.112
BiLSTM (Samaan et al., 2022)	0.76 ± 0.103	0.882 ± 0.091	0.949 ± 0.082	2.894 ± 2.717	0.76 ± 0.103	0.998 ± 0.001	0.722 ± 0.115
GRU (Samaan et al., 2022)	0.762 ± 0.091	0.891 ± 0.09	0.955 ± 0.077	3.092 ± 3.286	0.762 ± 0.091	0.998 ± 0.001	0.723 ± 0.105

Among all evaluated architectures, the Transformer and ResNet models achieved the highest overall performance. The Transformer obtained the best average Accuracy (0.828 ± 0.061), Top-3 Accuracy (0.942 ± 0.050), and Top-10 Accuracy (0.986 ± 0.017), together with an F1-Score of 0.792 ± 0.074 and low loss values (1.197 ± 0.732), indicating strong classification capability and stable behavior across runs.

The ResNet-based model achieved comparable performance, reporting an Accuracy of 0.823 ± 0.093, Top-3 Accuracy of 0.916 ± 0.083, and Top-10 Accuracy of 0.965 ± 0.058. It also obtained the lowest loss (1.195 ± 1.020) and a similarly high F1-Score (0.791 ± 0.104). Although the Transformer slightly outperformed ResNet in average metrics, this difference is largely influenced by a single low-performing outlier observed in the ResNet experiments. As illustrated in Figure 4, the dispersion of the ResNet results reveals consistently higher-performing validation cases, suggesting stronger generalization behavior across cross-validation folds. The high Specificity achieved by ResNet (0.998 ± 0.001) further indicates an excellent ability to correctly identify non-target classes while minimizing false positives.

The hybrid ResNet-Transformer architecture achieved intermediate performance, with an Accuracy of 0.782 ± 0.080 and an F1-Score of 0.738 ± 0.095. While combining convolutional feature extraction with self-attention improved performance over several recurrent baselines, the hybrid model did not surpass the standalone ResNet or Transformer architectures, suggesting that the additional architectural complexity did not translate into consistent gains for the keypoint-based representation.

The group of recurrent architectures proposed by Samaan et al. (2022)—namely LSTM, BiLSTM, and GRU—displayed competitive results, with accuracies above 0.76 and F1-Scores exceeding 0.72. Within this group, the GRU model achieved the highest Top-10 Accuracy (0.955 ± 0.077) and maintained a balanced performance across other metrics. The BiLSTM and LSTM variants achieved similar results, with minor differences in loss values and distributional stability.

The Simple RNN achieved moderate performance (Accuracy of 0.677 ± 0.106, F1-Score of 0.634±0.115), outperforming only the single-layer LSTM, which recorded the lowest scores in all metrics (Accuracy of 0.612±0.052, Top-3 Accuracy of 0.788±0.067, Top-10 Accuracy of 0.928±0.070, and F1-Score of 0.558± 0.062). These results suggest that architectures capable of modeling richer temporal or hierarchical representations generally yield superior performance in the isolated sign classification task.

Across all evaluated models, Specificity remained consistently high (≥0.997), indicating that false positives were rare regardless of the chosen architecture. However, differences in Accuracy, F1-Score, and loss values highlight that the selection of the model architecture plays a decisive role in optimizing recognition performance for this task.

Given the significant architectural variability among the evaluated models, including differences in network depth, parameterization, and computational complexity, their efficiency was additionally assessed in terms of training and inference times. As shown in Table 3, recurrent architectures generally required longer training periods. The proposed LSTM model exhibited the longest training time among recurrent approaches (739.667 ± 44.267,s), followed by the BiLSTM (Samaan et al., 2022) (526.303 ± 24.148,s) and the Simple RNN (303.277 ± 15.061,s). These results suggest that sequential processing and bidirectional temporal modeling increase computational cost during optimization. In contrast, the GRU (Samaan et al., 2022) (231.654 ± 12.664,s) and the LSTM variant proposed by Samaan et al. (2022) (249.011 ± 10.193,s) achieved shorter training times while maintaining competitive performance. The ResNet architecture (255.018 ± 17.176,s), benefiting from residual skip connections that facilitate gradient propagation, reported training times comparable to these efficient recurrent models.

**Table 3:** Results of the efficiency evaluation.

Model	Training time (s)	Inference time (s)
ResNet	255.018 ± 17.176	0.468 ± 0.023
Transformer	665.001 ± 46.778	1.937 ± 0.057
ResNet-Transformer	954.214 ± 44.253	2.266 ± 0.065
RNN simple	303.277 ± 15.061	0.556 ± 0.036
LSTM	739.667 ± 44.267	0.746 ± 0.055
LSTM (Samaan et al., 2022)	249.011 ± 10.193	0.616 ± 0.042
BiLSTM (Samaan et al., 2022)	526.303 ± 24.148	1.262 ± 0.062
GRU (Samaan et al., 2022)	231.654 ± 12.664	0.615 ± 0.026

The attention-based architectures exhibited substantially higher computational demands. The Transformer required a training time of 665.001 ± 46.778,s, while the hybrid CNN + Transformer model recorded the longest overall training time (954.214 ± 44.253,s), reflecting the added complexity introduced by combining convolutional feature extraction with multi-head self-attention mechanisms.

Regarding inference efficiency, similar trends were observed. The CNN + Transformer architecture presented the slowest inference time (2.266 ± 0.065,s), followed by the Transformer (1.937 ± 0.057,s) and the BiLSTM (Samaan et al., 2022) (1.262 ± 0.062,s). In contrast, the GRU (Samaan et al., 2022) (0.615 ± 0.026,s) achieved inference times comparable to the LSTM (Samaan et al., 2022) (0.616 ± 0.042,s) and the standard LSTM (0.746 ± 0.055,s). The Simple RNN showed faster inference (0.556 ± 0.036,s), while the ResNet architecture achieved the shortest inference time overall (0.468 ± 0.023,s), demonstrating the computational efficiency of convolutional processing with residual connections.

Although the Transformer model achieved highly competitive classification results, its computational cost was considerably higher than that of ResNet. Specifically, the Transformer required approximately three times longer training time and nearly five times longer inference time compared to the ResNet architecture, despite producing comparable performance metrics. Considering the trade-off between predictive performance and computational efficiency, the ResNet model was therefore selected as the best-performing architecture and subsequently subjected to hyperparameter optimization using Optuna.

### 4.2 Hyperparameter optimization results

Following the optimization procedure described in Section 3.4, this section presents the analysis and outcomes of the hyperparameter search conducted on the selected 1D ResNet architecture. The objective of this stage was not only to identify the configuration achieving the highest predictive performance, but also to analyze the optimization dynamics and determine which architectural and training parameters most strongly influenced model generalization under subject-independent evaluation.

**Figure 5:**
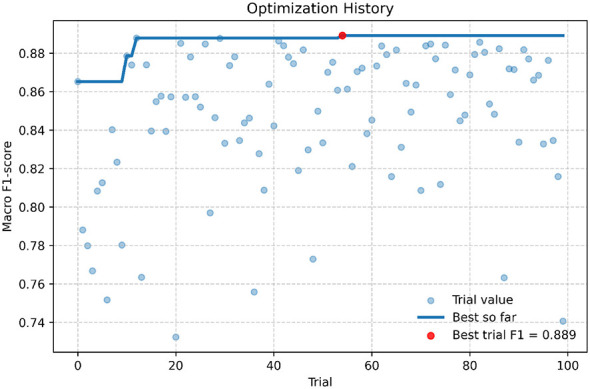
Hyperparameter optimization trajectory for the ResNet model.

Figure 5 illustrates the optimization trajectory obtained during the Optuna search process, including individual trial performances and the evolution of the best-so-far score. Competitive configurations were identified early in the search, with the first trial already achieving a macro *F*_1_-score above 0.86, indicating an adequately defined search space. Performance improved progressively during the initial exploration phase, reaching values close to 0.88 around trial 10, while trial 12 marked the first configuration surpassing this threshold. After this stage, the optimization exhibited higher variability as diverse architectural configurations were explored. The best-performing configuration was obtained at trial 54, achieving a macro *F*_1_-score of 0.889. Although subsequent trials occasionally exceeded 0.88, none approached the performance of trial 54, suggesting convergence toward a near-optimal region of the search space. Overall, most performance gains occurred during the early and mid stages of optimization, whereas later trials primarily confirmed the stability of the discovered optimum rather than yielding substantial improvements.

**Figure 6:**
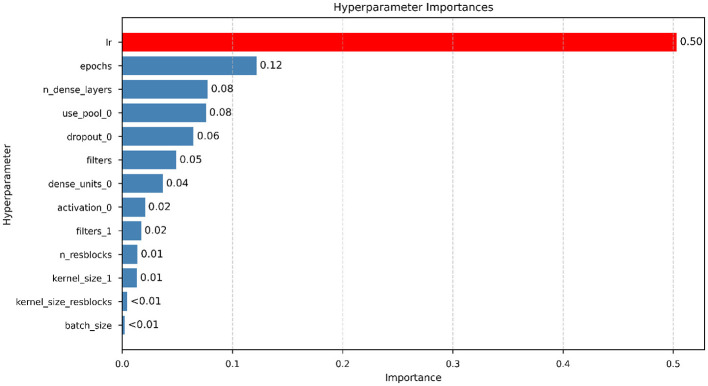
Hyperparameter importance analysis for the ResNet model.

The relative importance of each hyperparameter was analyzed using Optuna's parameter importance estimation (Figure 6). The results indicate that optimization performance was primarily driven by training dynamics and early feature extraction decisions rather than deeper architectural variations. The learning rate emerged as the dominant factor, accounting for approximately 0.50 of the total importance, highlighting its critical role in convergence stability and generalization during gradient-based optimization. Among architectural parameters, the use of pooling in the first residual block (*use*_*pool*_0) and the number of dense layers (*n*_*dense*_*layers*) showed notable influence (both ≈0.08), followed by dropout in the first dense layer (*dropout*_0, ≈0.06) and the number of filters within residual blocks (*filters*, ≈0.05), suggesting that performance depends on an appropriate balance between feature compression, representational capacity, and regularization.

Parameters associated with early feature extraction, including the number of filters in the initial convolution (*filters*_1) and the activation of the first dense layer (*activation*_0), exhibited smaller contributions (≈0.02), while kernel size of the first convolution and the number of residual blocks showed marginal influence (≈0.01). Conversely, batch size and residual kernel size presented negligible importance (< 0.01); however, the low contribution of batch size should be interpreted cautiously due to the restricted search range (32–64), indicating robustness to small batch variations rather than intrinsic irrelevance. The analysis suggests that performance gains were mainly governed by optimization-related parameters and early representational choices within the explored search space.

The optimal hyperparameter configuration identified during the optimization process is summarized in Table 4. The resulting architecture reflects a balanced design combining moderate initial feature extraction capacity with deeper residual representations and a lightweight classification head. In particular, the optimized model employs three residual blocks with 128 filters, a larger initial temporal receptive field (*kernel*_*size*_1 = 5), and a single dense classification layer composed of 64 units with ELU activation and minimal dropout regularization. The selected learning rate (3.22 × 10^−4^) and batch size (64) further support stable convergence, consistent with the importance analysis highlighting optimization dynamics as the primary performance driver.

**Table 4:** Optimized configuration of the ResNet model obtained after hyperparameter optimization.

Component	Parameter	Value
Global	Filters_1	32
	Filters	128
	Kernel_size_1	5
	Kernel_size_resblocks	4
	n_resblocks	3
	n_dense_layers	1
	lr	0.000322
	Batch_size	64
	Epochs	50
Residual blocks	Block 1 pooling	False
	Block 2 pooling	False
	Block 3 pooling	True
Dense layer 1	Units	64
	Activation	Elu
	Dropout	0.015941

To evaluate the robustness of the optimized configuration, the model was assessed using a Leave-One-Subject-Out cross-validation protocol. The detailed results are presented in Table 5. Across all subjects, the optimized model demonstrated consistently high performance, achieving accuracies above 0.83 in all folds and exceeding 0.95 in several cases. The macro *F*_1_-score ranged from 0.778 to 0.948, indicating strong subject-independent generalization despite inter-subject variability. Notably, specificity remained close to 1.0 across all evaluations, confirming a low false-positive rate, while training and inference times remained stable between folds. These results demonstrate that the hyperparameter optimization process not only improved peak performance but also enhanced model stability and generalization across unseen subjects.

**Table 5:** Cross-validation performance of the optimized ResNet model under leave-one-subject-out evaluation.

Test subject	Training time (s)	Inference time (s)	Epochs	Loss	Accuracy	Top-3 Acc.	Top-10 Acc.	Sensitivity	Specificity	F1-score
1	89.976	0.761	11	0.458	0.913	0.966	0.987	0.911	0.999	0.891
2	134.657	0.703	17	1.105	0.831	0.872	0.936	0.822	0.999	0.79
3	156.887	0.712	20	0.17	0.951	0.994	1.0	0.957	1.0	0.941
4	141.83	0.71	18	0.2	0.932	0.997	1.0	0.936	0.999	0.921
5	64.246	0.691	8	0.405	0.895	0.975	0.976	0.89	0.999	0.862
6	87.687	0.696	11	0.242	0.943	0.984	0.987	0.948	1.0	0.935
7	125.46	0.69	16	0.165	0.959	0.991	0.992	0.958	1.0	0.948
8	125.882	0.69	16	0.629	0.838	0.962	0.995	0.821	0.999	0.778
9	72.204	0.701	9	0.452	0.891	0.968	0.985	0.891	0.999	0.865
10	147.849	0.673	19	0.41	0.92	0.978	0.988	0.916	0.999	0.901

**Figure 7:**
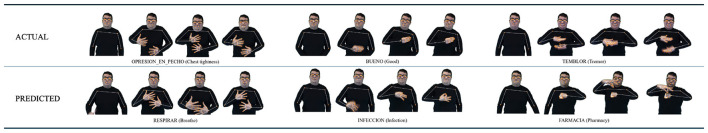
Examples of model misclassifications.

After analyzing the predictions of the best-performing model, the proportion of misclassified examples was calculated with respect to the total instances of each real class, and some examples are shown in Figure 7. The class that exhibited the greatest difficulty in recognition was OPRESION_EN_PECHO, which was systematically confused with the class RESPIRAR on 50 occasions, corresponding to 100% of the cases. Although both are performed with two hands placed on the chest, the key distinction lies in the movement: in RESPIRAR, the hands simulate an expansion gesture, as if the lungs were inflating during inhalation, whereas in OPRESION_EN_PECHO the hands represent strong pressure on the chest, typically accompanied by a facial expression of discomfort.

On the other hand, the model exhibited a 64% misclassification rate for the class BUENO (32 misclassified samples), which was confused with the class INFECCION. Similar to the previous case, both signs share similarities in the number of arms involved and in the location where they are performed. However, the manual configuration differs in key aspects: in INFECCION, the index finger is flexed so that the fingertip touches the thumb, forming a shape similar to the F handshape in LSM; whereas in BUENO, only the thumb is flexed inward toward the palm, corresponding to the B handshape in LSM.

Another example of confusion related to hand configurations is observed in the classes TEMBLOR and FARMACIA, where 26 samples were misclassified, representing 52% of the total. The main similarity between these two signs, as in previous cases, is that both are performed with two hands and involve movements prone to information loss. This is due to both the position of the hands and the speed of the movement, which generates motion blur in the image and hinders the detection of manual features.

Despite the observed confusions, our best model proves to be robust to subtle distinctions in most cases within the corpus. It also demonstrates the ability to learn semantic differences inherent to the signs, successfully identifying related gestures—for example, head direction—which highlights its sensitivity to certain non-manual features. Furthermore, the model performs well on less ambiguous signs, particularly those with clear differences in spatial position or manual configuration, where it achieves successful generalization. The complete list of correctly and incorrectly classified instances is presented in Supplementary Table 1 in the Supplementary material.

## 5 Conclusions

This work presents relevant advances in the field of automatic sign language recognition. First, a sign language corpus was designed and developed, validated by experts from an association of the deaf community, ensuring the pertinence and representativeness of the collected data. Based on this corpus, RGB video data were recorded and features were extracted to train different neural network models, among which the ResNet architecture demonstrated outstanding performance. Following the comparative evaluation, a hyperparameter optimization stage was conducted to further refine the selected model. The optimization analysis revealed that performance improvements were driven primarily by training dynamics, generalization capacity, and regularization strategies rather than by increasing architectural depth alone. In particular, optimization-related parameters such as the learning rate, pooling configuration, and regularization mechanisms showed greater influence than deeper structural variations, highlighting the importance of stable training behavior over model complexity. Finally, the optimized model achieved an F1-score of 94%, reflecting a strong balance between precision and recall and confirming the effectiveness and robustness of the proposed approach for subject-independent sign language recognition.

In comparison with the state of the art, most existing corpora are characterized by a limited number of signs to be recognized and by the inclusion of only a few participants, typically no more than five. In contrast, our study comprises more than 100 signs and incorporates greater subject variability with a total of 10 non-expert participants. This increase in problem complexity is reflected in the results: although the metrics achieved are lower than those reported in previous works, the evaluation under a more challenging scenario provides a more realistic and representative assessment of the model's performance.

Among the main limitations identified, the model shows sensitivity to motion blur, as rapid gestures reduce the quality of the keypoints and lead to systematic recognition errors. The model also struggles to discriminate fine-grained manual configurations, such as finger flexion in partially occluded positions, which highlights limitations in hand representation. In addition, a strong reliance on manual features was observed, underscoring the need to incorporate more complex non-manual characteristics, such as facial expressions. Finally, the clear influence of morphological factors on the model's generalization ability suggests the importance of increasing both the diversity and the number of participants involved in the corpus collection. These limitations were considered a priority to be addressed in future work.

The next step is to advance toward continuous sign recognition with the aim of generating complete sentences in Spanish, a considerably more challenging scenario due to the complexity of sign transitions and the need to model richer temporal dependencies. Likewise, it is essential to expand the corpus not only in terms of the number of participants but also by incorporating a wider variety of contexts, which would enable the scaling of the resource toward more robust translation applications. However, the current recognition paradigm faces an inherent limitation regarding scalability: As the number of classes increases, the classification task becomes more challenging due to the increased decision space. In this context, it will be necessary to explore the development of recognition methods that are strongly stratified and grounded in the semantic and linguistic foundations of LSM. Such an approach would not only improve scalability but also facilitate the extension of the methodology to other sign languages, paving the way for multilingual sign language recognition models that foster broader and more integrated understanding across different deaf communities worldwide.

## Data Availability

The datasets presented in this study can be found in online repositories. The names of the repository/repositories and accession number(s) can be found at: https://doi.org/10.5281/zenodo.18330565 and https://github.com/XxANTONIO73xX/LSM-DynamicSigns-DeepLearning.
