# Supplementary material for: Automatic recognition of dynamic signs of Mexican sign language using deep learning
Source: Front Artif Intell. 2026 Apr 16;9:1794923. doi: 10.3389/frai.2026.1794923 (PMC13128546; doi:10.3389/frai.2026.1794923)
Supplement: Supplementary file 1 [file Data_Sheet_1.pdf]

# Supplementary Material

## 1 TASK EVALUATION METRICS

To assess the performance of the proposed models, we employed a set of widely adopted evaluation metrics in machine learning, derived from the confusion matrix and probability-based predictions. These metrics allow a comprehensive analysis of classification performance, both at the individual prediction level and in terms of probabilistic confidence.

$$\text{Accuracy} = \frac{TP + TN}{TP + TN + FP + FN} \tag{S1}$$

Accuracy measures the overall proportion of correctly classified instances. However, it may be misleading in highly imbalanced datasets, where the majority class dominates the metric.

$$\text{Top-}k\text{ Accuracy} = \frac{1}{N} \sum_{i=1}^{N} 1\{y_i \in \hat{Y}_i^{(k)}\}, \tag{S2}$$

where $\hat{Y}_i^{(k)}$ denotes the set of the $k$ most probable predictions for instance $i$, and $1\{\cdot\}$ is the indicator function. This metric is particularly relevant in tasks where multiple plausible predictions exist, such as sign language recognition.

$$\mathcal{L}_{CCE} = -\frac{1}{N} \sum_{i=1}^{N} \sum_{c=1}^{C} y_{i,c} \log(\hat{p}_{i,c}) \tag{S3}$$

where $y_{i,c} \in \{0, 1\}$ is the one-hot encoded ground truth label for class $c$, and $\hat{p}_{i,c}$ represents the probability predicted by the model for class $c$ in sample $i$. The categorical cross-entropy loss evaluates how well the predicted probability distribution aligns with the true labels.

$$\text{Sensitivity (Recall)} = \frac{TP}{TP + FN} \tag{S4}$$

Recall quantifies the model's ability to correctly identify positive samples.

$$\text{Specificity} = \frac{TN}{TN + FP} \tag{S5}$$

Specificity complements recall by measuring the proportion of true negatives correctly identified.

$$\text{F1-Score} = 2 \cdot \frac{\text{Precision} \cdot \text{Recall}}{\text{Precision} + \text{Recall}} \tag{S6}$$

The F1-score balances precision and recall, providing a robust measure in scenarios with class imbalance, where both false positives and false negatives must be penalized symmetrically, with

$$\text{Precision} = \frac{TP}{TP + FP}. \tag{S7}$$

## 2 DETAILED CLASSIFICATION RESULTS

In this section, we provide the exhaustive list of glosses evaluated in the Top-1 classification task. Table S1 details the performance of the optimal ResNet model, categorizing glosses that achieved perfect classification (1.0) and those that did not (<1.0).

**Table S1.** Top-1 classification results by the optimal ResNet model.

| **Correctly classified glosses (1.0):** |
|---|
| ACCIDENTE, AHI, AHORA, AMBULANCIA, AMIGO, ANTES, ARTICULACIONES, AYUDA, BAÑO, BOMBEROS, BONITO, BRAZO, BRAZO_HINCHADO, BRAZOS, CABEZA, CALIENTE, CANCER, CARRO, CARTERA, CASA, CITA, COMIDA, COMO, COMPRAR, CONOCER, CORAZON, CUANTO, CUERPO, CUERPO_CORTADO, CUIDAR, DAÑAR, DESLIZAR_EN_CUERPO, DESMAYAR, DIA, DIARREA, DIFICIL, DINERO, DOCTOR, DOLOR, DONDE, EDIFICIO, EL, EMBARAZADA, ENFERMERO, ENFERMO, EPILEPSIA, ESPAÑOL, ESPECIAL, ESTAR, ESTO, ESTOMAGO, EXPLOSION, FARMACIA, FIEBRE, FUEGO, GARGANTA, GOLPE, GRACIAS, GRIPE, GUSTAR, HABER, HERIDA, HOLA, HOSPITAL, INFECCION, IR, LLAMAR, LSM, MAL, MAREADO, MEDICINA, MI, MUCHO, NADA, NEUTRO, NO, NO_ENTENDER, NO_ESCUCHAR, NO_PODER, NOCHE, NOS_VEMOS, OTRA_VEZ, PALPITACION, PERDER, PIERNA, POLICIA, POR_FAVOR, PRESION_ARTERIAL, PRESIONAR, PROXIMO, PULMONES, QUIMICOS, RESPIRAR, ROBAR, ROSTRO, ROSTRO_HINCHADO, SENTIDO_DEL_GUSTO, SENTIR, SI, SORDO, SU, SUCESO, TARDE, TELEFONO, TENER, TOS, TRABAJO, URGENCIA, VOMITO, VOZ, YO |
| **Misclassified glosses (<1.0):** |
| BUENO, CORTAR_ABRIR, DIABETES, ELEVADO, MANEJAR, NECESITAR, NO_NADA, OPRESION_EN_PECHO, PLATICAR, TEMBLOR, TEMPERATURA |
